# The impact of COVID-19 outbreak on otolaryngology practice, jordanian experience: A qualitative study

**DOI:** 10.1016/j.amsu.2021.01.066

**Published:** 2021-01-29

**Authors:** Ahmad Al Omari, Yazan Kanaan, Amjad Nuseir, Ra'ed Al-ashqar, Hasan Al-Balas, Osama Hamarneh, Firas Alzoubi

**Affiliations:** a Otolaryngology Department, Faculty of Medicine, Jordan University of Science and Technology, P O Box 3030, Irbid 22110, Jordan; b Otolaryngology Department, Faculty of Medicine, Yarmouk University, Jordan; c Otolaryngology Consultant, Al Abdali Hospital, Amman, Jordan

**Keywords:** Coronavirus, COVID-19, Infection, Pandemic, Otolaryngology, Lockdown

## Abstract

**Background:**

In response to the rapid spread of coronavirus disease 2019 (COVID-19) caused by “severe acute respiratory syndrome coronavirus 2” (SARS-CoV-2), many countries including Jordan have implemented strict lockdowns. These lockdowns were associated with temporary suspension of all outpatient clinics and all elective (Non emergent, non-oncologic) surgical procedures.

**Objective:**

We aimed to report the impact of COVID-19 outbreak on otolaryngology practice in Jordan.

**Methods:**

Retrospectively we reviewed all admissions to the otolaryngology wards of King Abdullah University Hospital during the lockdown and for the same dates for the year 2019, results were compared.

Additionally, an online questionnaire was sent to a sample of Jordanian otolaryngologists in June 2020. The questionnaire was comprised of a series of multiple choice questions regarding each physician's participation in the treatment or screening of COVID-19 patients, the number of consultations during the lockdown, the numbers of elective and emergency surgical procedures performed during the lockdown and the effects the lockdown had on their practices, their patients conditions and teaching and training processes.

The study was done in line with the criteria set by the Standards for Reporting Qualitative Research (O’Brien et al., September 2014) [12].

**Strengths and weaknesses:**

In our study, we aimed to include the experience of all otolaryngology practitioners in Jordan, providing a comprehensive view of the lockdown effects on practice in the region. The data found is likely representative of lockdown effects on all departments, not just otolaryngological practice, and may be beneficial in providing a pathway to minimize any negative impact on patient care.

However, our data may be limited due to its dependence on responses through a Whatsapp questionnaire, with no guarantee that the answers provided are fully accurate. It also may have a certain degree of sampling bias, as while the questionnaire was sent to all ENT practitioners in Jordan, answering it was totally optional, and so people who did not respond to the survey were not accounted for.

**Results:**

During the lockdown period in Jordan all outpatient clinics were closed, and all elective surgical procedures (non-emergency and non-oncologic procedures) were suspended. During the lockdown it was observed that there was a reduction in the number of admissions related to post-operative complications, head and neck abscesses & infections and foreign bodies related admission when compared to the same period of 2019.

A total of 144 otolaryngologists have participated in the questionnaire part of the study. More than half of the participants (n = 80, 55.6%) reported providing 10 or less consultations during the lockdown, more than half of the them have not performed any emergency surgical procedures during the lockdown, and a total of 110 (76.4%) of the 144 participants reported having at least 1 patient whose condition worsened during the lockdown due to lack or delay in medical care.

**Conclusion:**

The COVID-19 pandemic, and the resultant lockdown period in Jordan has caused a significant shift in otolaryngological practice throughout the country, with a complete cessation of all outpatient clinics and elective surgical procedures and admissions, with activity being limited to oncological and emergency procedures only. These changes have already impacted the dynamics of patient care and might lead to a risk of diagnostic delays which will have severe impacts on patient's health.

## Highlights

• SARS-CoV-2 is a highly contagious virus and can spread through both direct means (droplet and human-to-human transmission) and by indirect contact (contaminated objects and airborne contagion).
• In response to the rapid spread of coronavirus disease 2019 (COVID-19) caused by “severe acute respiratory syndrome coronavirus 2” (SARS-CoV-2), many countries including Jordan have implemented strict lockdowns
• The COVID-19 pandemic, and the resultant lockdown period in Jordan has caused a significant shift in otolaryngological practice worldwide

## 1 Introduction

In December 2019, a pneumonia outbreak associated with a new coronavirus was reported in Wuhan, Hubei province, China [1]. This disease was named coronavirus disease 2019 (COVID-19) by the World Health Organization (WHO). The causative virus was called “severe acute respiratory syndrome coronavirus 2” (SARS-CoV-2) by the international committee of the Coronavirus Study Group (CSG) [2]. The situation report issued by the WHO on December 15, 2020 listed more than 70 million confirmed COVID-19 cases and around 1.6 million deaths worldwide [3].

SARS-CoV-2 is a highly contagious virus and can spread through both direct means (droplet and human-to-human transmission) and by indirect contact (contaminated objects and airborne contagion). There is no specific treatment for COVID-19 disease up to now [4].

On March 2, 2020, the Prime Minister of Jordan reported the first case of COVID-19 in Jordan [5]. On March 13, a wedding ceremony led to a large outbreak of COVID-19 in northern Jordan, 76 of the 350 attendees were diagnosed with COVID-19 [6]. Till June 17, 2020 a total of 981 COVID-19 cases and 9 COVID-19 related deaths were confirmed in Jordan [3].

In response to the rapid spread of the virus around the world on March 14, 2020 the Government of Jordan suspended schools and universities and banned public gatherings including prayers in mosques and churches despite only having 1 confirmed COVID-19 patient.

On March 17, 2020, all flights to and from Jordan were suspended [7]. On March 20, 2020, the Government of Jordan announced a nationwide strictly enforced curfew beginning March 21. The curfew prohibited the movement of people and closed all shops [8]. This curfew lasted for 4 days but then was gradually relaxed and people were allowed to walk to buy groceries from local stores from 10 a.m. till 6 p.m. For a total of 6 weeks the lockdown included a ban on the use of cars except for health care providers and essential sector workers. During the lockdown any patient with an emergency requiring evaluation in a hospital had to contact the civil defense in order to be transported to the nearest hospital by an ambulance.

King Abdullah University Hospital (KAUH); a tertiary care hospital in the north of Jordan that is affiliated with Jordan University of Science and Technology was the primary center for treating and isolating COVID-19 patients in the north of Jordan. From the first day of the lockdown [March 21, 2020] till May 30, 2020, as per hospital's regulations, all outpatient clinics were temporarily suspended including otolaryngology clinics; diagnostic and therapeutic procedures were limited to emergency and oncology cases only. All elective [Non-emergent & non-oncologic] surgical procedures were postponed.

The aim of this study is to report the impact of COVID-19 outbreak on otolaryngology practice in Jordan.

## 2 Methods

### 2.1 Our Institution's experience

In this study we retrospectively reviewed all admissions to the otolaryngology wards of King Abdullah University Hospital (many of which we had the role of attending physician for), from the first day of the lockdown [March 21, 2020] till May 30, 2020, during which all outpatient clinics were suspended and only oncology & emergency cases were admitted.

After being granted permission by the institutional review board (no 413–2020) all inpatient medical records were retrospectively reviewed. Particular attention was given to the number and type of procedures performed during the pandemic. We retrospectively reviewed all admissions to our wards for the same period of the year 2019 for comparison.

Admission were divided into 3 categories:

• **Emergency** surgical and non-surgical admissions: this category included emergency post-operative complications such as post-operative bleeding or infections, foreign bodies impaction either in the upper aerodigestive tract, nose or ear requiring examination or removal under general anesthesia, airway emergencies requiring medical management, tracheostomies or tracheal dilatation, infections requiring intravenous antibiotics or abscesses drainage and vestibular neuritis.
• **Oncological surgical admissions:** such as diagnostic biopsies, total laryngectomies, neck dissections, thyroidectomies, laser resection of laryngeal cancers and lip cancer resection.
• **Elective surgical admissions:** Other than emergency and oncologic surgical admissions, this category included a wide variety of nasal, ear, head & neck, laryngeal and minor surgical procedures.
  o **Nasal surgeries** included endoscopic nasal surgeries (Including endoscopic sinus surgeries, turbinate reduction, endoscopic dacrocystorhinostomy procedures and cerebrospinal fluid leak repair), septoplasty and rhinoplasty procedures.
  o **Ear surgeries** included tympanoplasty surgeries, mastoidectomy procedures, cochlear implants, stapedotomy and otoplasty procedures
  o **Head and neck surgeries** included parotid surgeries, thyroid surgeries, total laryngectomies, neck dissections and others.
  o **Laryngeal surgeries** included diagnostic microlaryngoscopy procedures, removal of laryngeal nodules or polyps and others.
  o **Minor surgeries** included tonsillectomies, adenoidectomies, and grommet tubes insertion.

### 2.2 Jordanian otolaryngologists’ perspective

An online questionnaire using Google Forms was used to collect data from otolaryngology physicians working in Jordan from all sectors (University hospitals, military hospitals [Royal Medical Services], ministry of health or private sector) to see the effects of the lockdown on their practices and the rules they've played during the pandemic. The questionnaire was sent to otolaryngology physicians on June 1st, 2020 through Whatsapp groups. To maintain the privacy and confidentiality of all data collected the questionnaires were anonymous. Ethical approval was obtained from the Institutional Review Board.

In order to minimize sampling and response bias, the questionnaire was sent to all known registered ENT practitioners in Jordan, and their participation was non-compulsory. However, given the impersonal nature of Whatsapp, there may be bias in who chooses to respond to the questions and who does not that we cannot fully account for, possibly skewing data. There may also be ENT practitioners unaccounted as they are not registered or did not receive the questionnaire.

The questionnaire was in English, contained a series of multiple-choice questions regarding each physician's participation in the treatment or screening of COVID-19 patients, the number of consultations during the lockdown, the numbers of elective and emergency surgical procedures performed during the lockdown and the effects of the lockdown on their practices and their patients conditions. The questions were generated based on what we predicted would be the most affected areas of practice, and were designed in order to zero in on the effects each practitioner experienced due to the lockdown, and to gather calculable data from their answers.

## 3 Results

### 3.1 Our Institution's experience

At our otolaryngology unit a total of 154 elective surgical procedures were postponed during the period of the lockdown. Only oncological & emergency surgical procedures were performed. A total of 10 patients underwent surgical procedures which included 3 emergency airway surgical procedures (3 tracheostomies) and 6 oncological procedures (2 Thyroidectomies, 1 microlaryngoscopy & biopsy, 1 laser-assisted cordectomy for laryngeal cancer, 1 tongue base mass biopsy and 1 lip cancer excision with neck dissection). Additionally 4 emergency cases were admitted to our wards but didn't require any surgical intervention and were treated medically, these included a case of peri-orbital cellulitis treated with intravenous antibiotics, a case of acute laryngitis, a case of acute tonsillitis with a decrease in oral intake treated with intravenous antibiotics and fluids and a case of esophageal food impaction that was treated conservatively.

Between March 21 and May 30 of 2019 a total of 235 elective, 12 oncological and 14 emergency surgeries were performed.

**Fig. 1:**
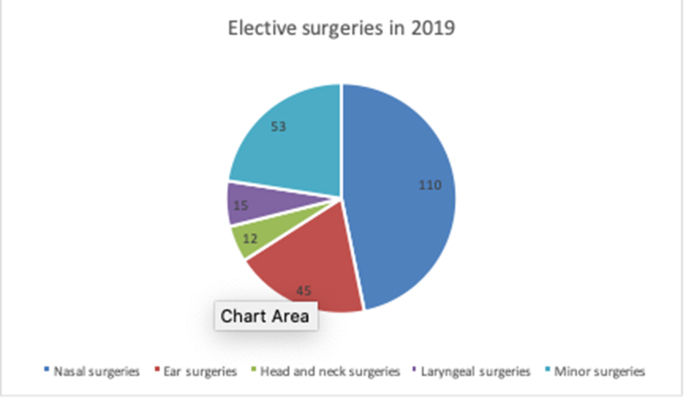
Summary of elective surgeries performed at our unit from March 21, 2019, till May 30, 2019.

The elective surgeries in this period of 2019 are summarized in Fig. 1.

The oncological procedures in 2019 were as follows: 1 total laryngectomy with neck dissection and 11 diagnostic biopsies; 6 post-nasal space masses biopsies, 3 diagnostic microlaryngoscopy with biopsy procedures, 1 excisional lymph node biopsy and 1 biopsy from a laryngectomy stoma for recurrent laryngeal cancer. This was compared to 7 oncological procedures performed during the lockdown, of which were only 2 diagnostic biopsies.

**Fig. 2:**
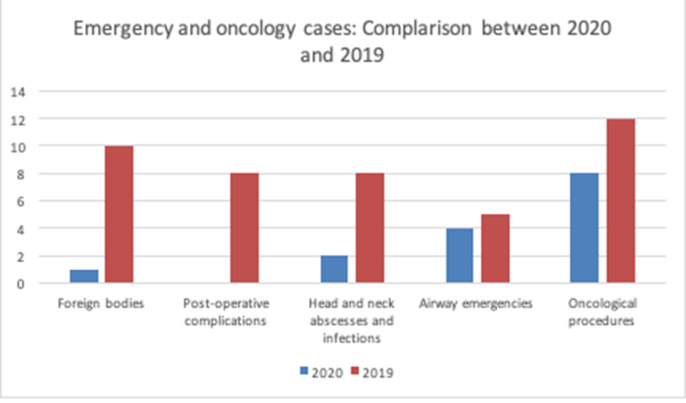
Comparison between emergency and oncological admissions to our words during the lockdown and the same period of 2019.

Of a total of 32 emergency cases cared for by our team, 17 underwent surgery. Of those, 3 were tracheostomies, 10 foreign body removals, 3 abscess drainage (2 septal abscess post trauma, 1 peritionsillar abscess), and 1 subglottic stenosis dilatation. Additionally, 15 patients with emergency conditions were admitted to our wards and treated medically, 8 of which had post-operative complications (4 post tonsillectomy patients with decreased oral intake or post tonsillectomy bleeding, 1 case of epistaxis post septoplasty, 1 infection post endoscopic sinus surgery, 1 case of hypocalcemia post parathyroid adenoma excision, and 1 wound infection post total laryngectomy), 6 cases of infection for IV antibiotics (1 case of facial cellulitis, 1 case of otitis media, 3 cases of follicular tonsillitis), and 1 case of stridor. Fig. 2 compares the emergency and oncology admissions between 2019 and 2020 for the same period.

### 3.2 Jordanian otolaryngologists’ perspective

A total of 144 otolaryngologists have participated in the study: 102 (70.8%) specialists and 42 (29.2%) residents. 70 (48.6%) of the participants work in the private sector, 28 (19.4%) in the ministry of health, 24 (16.7%) in university hospitals and 22 (15.3%) in military hospitals.

A total of 31 (21.5%) of the 144 participants reported being directly involved in COVID-19 patients’ treatment, and 47 (32.6%) reported being part of COVID-19 screening teams.

Table 1 shows details of the practices of Jordanian otolaryngologists during the lockdown. More than half of the participants (n = 80, 55.6%) reported providing 10 or less consultations during the lockdown and 28 (19.4%) of participants reported providing more than 30 consultations. These consultations were provided either remotely through online messages or phone calls, or directly face to face with patients. More than half of them (n = 86, 59.7%) reported not performing any elective surgeries during the lockdown, only 6 (4.2%) reported performing more than 10 elective surgical procedures during the lockdown. More than half of the participants have not performed any emergency surgical procedures during the lockdown, and a total of 61 (42.4%) reported performing 1–5 emergency surgical procedures during the lockdown.

**Table 1:** Details of the practices of Jordanian otolaryngologists during the lockdown.

Table 1		Physicians, n (%)
**Number of scheduled consultations**	*0*	35 (24.3)
	*1–10*	45 (31.3)
	*11–20*	25 (17.4)
	*21–30*	11 (7.6)
	*> 30*	28 (19.4)
**Number of elective surgeries during the lockdown**	*0*	86 (59.7)
	*1–10*	52 (36.1)
	*11–20*	0 (0)
	*21–30*	1 (0.7)
	*> 30*	5 (3.5)
**Number of emergency surgical procedures during the lockdown**	*0*	73 (50.7)
	*1–5*	61 (42.4)
	*6–10*	9 (6.3)
	*> 10*	1 (0.7)

Table 2 summarizes the responses of the participants to questions regarding the effects of the lockdown on their practices and their patients’ condition. A total of 110 (76.4%) of the 144 participants reported having at least 1 patient whose condition worsened during the lockdown due to lack or delay in medical care, and 6 physicians reported having at least 1 mortality during the lockdown due to lack or delay in medical care.

**Table 2:** Summary of the responses of the participants to questions regarding the effects of the lockdown on their practices and their patients’ condition.

Table 2		Physicians, n (%)
**Number of patients whose conditions worsened during the lockdown due to lack or delay in medical care**	*0*	34 (23.6)
	*1–5*	56 (38.9)
	*6–10*	21 (14.6)
	*11–20*	12 (8.3)
	*> 20*	21 (14.6)
**Number of mortalities during the lockdown due to lack or delay in medical care**	*0*	138 (95.8)
	*1–5*	6 (4.2)
	*> 5*	0 (0)

## 4 Discussion

During the lockdown period in Jordan between March 21st and May 30th, 2020, the otolaryngology department of KAUH underwent several changes as per hospital regulations in response to the COVID-19 pandemic, including the closure of all outpatient clinics, and suspension of all elective surgical procedures (non-emergency and non-oncologic procedures). These measures led to a significant change in otolaryngological practice for this period in comparison to the same period in 2019.

A total of 154 elective surgical procedures were postponed during the lockdown, while in 2019 in the same time period 235 elective surgical procedures were performed, with the difference probably being due to the lack of new elective cases being scheduled through the outpatient clinics. When comparing data for post-operative complications between 2019 and 2020, we see there are no cases admitted with post-operative complications during the lockdown period in 2020, while there are 8 admissions during this same period in 2019. This drastic difference is probably simply due to the fact that all elective surgeries were cancelled during the lockdown period, thus there were far fewer operations performed in comparison to 2019. Similarly, while in 2019 there were 10 emergency admissions for foreign body aspiration/ingestion removal, all from the pediatric population, in the same period in 2020, there was only 1. This could be explained by the fact that during the lockdown period, most parents were quarantined at home with their children, thus leading to extra care and watchfulness of the child.

There was also a reduction in cases of head and neck abscesses and infections, with 8 cases admitted in 2019 vs only 2 in the same period in 2020. This reduction could be in part due to the lack of interpersonal contact during the lockdown period in 2020 (reducing the number of cases of tonsillitis and peritonsillar abscesses). Additionally, the restrictions on driving during the lockdown period likely led to a reduction in road traffic accidents, possibly explaining why in 2019 there were cases of septal abscesses post trauma while in 2020 there were none.

On comparing the oncological procedures performed during the lockdown period in 2020 to the same period in 2019, there is a small reduction in admissions from 12 in 2019 to 8 in 2020. As oncological procedures were not postponed during the lockdown, this difference is probably owing to the fact that there were fewer new oncological cases diagnosed as the outpatient clinics were suspended.

It is also worth mentioning, that in all the cases listed above, a possible reason for the decrease in admitted cases could be due to the fact that KAUH is a tertiary hospital situated on the outskirts of Ramtha city, and is considered isolated from the most populated areas in the north of Jordan, thus making access to the hospital more difficult during the lockdown period with restrictions on driving, with most emergency cases likely being taken to the nearest local facility by civil defense.

Contrastingly, the comparison between 2019 and 2020 for airway emergency procedures performed showed no significant difference, with 3 tracheostomies performed in both years during the same period, likely because, most commonly, tracheostomy is indicated because of prolonged intubation, the number of cases of which were unaffected by the lockdown.

Similar findings were found among otolaryngologists throughout Jordan as seen in the results of our questionnaire, with over half of the participants not having performed any emergency surgical procedures during the lockdown, and around 42.4% only performing 1–5 procedures during this period. These findings are likely mirrored worldwide in places where a full lockdown has taken place by healthcare personnel of all specialties. A study in India reports that the majority of ophthalmologists in India were not seeing any patients during their lockdown period, with a total cessation of elective surgeries and a significant reduction in emergency surgeries being performed [9]. Likewise, a study in the UK showed that during the national lockdown, there was a dramatic reduction in orthopedic trauma admissions [10], with similar results reported by the British Orthopedic Association in Glasgow [11].

It is important to note that, while 76.4% of the participants of the questionnaire reported having at least 1 patient whose condition worsened due to the delay or lack of medical care during the lockdown period, this is a subjective matter based on the opinion of the participants. The true extent of the effects of the lockdown period on patient care, morbidity and mortality is not yet apparent, and will have to be watched closely and studied retrospectively as we begin to reopen our clinics and resume patient follow up in the coming months.

## 5 Conclusion

The COVID-19 pandemic, and the resultant lockdown period in Jordan has caused a significant shift in otolaryngological practice throughout the country, with a complete cessation of all outpatient clinics and elective surgical procedures and admissions, with activity being limited to oncological and emergency procedures only. These changes have already impacted the dynamics of patient care as described above, yet we must remain watchful as the months progress in order to truly grasp the extent of the effects this lockdown has had on patient care in the long term, and react to these changes accordingly.

In light of these changes to practice, many months on, new practices have been adapted to minimize any negative effects to patient care. First of all, rather than complete closure of outpatient clinics and cessation of elective surgeries, these functions were kept active more recently, but at reduced capacity and with a multitude of protective measures in place to minimize risk to patients and healthcare staff. Furthermore, the Jordanian government is actively working on opening multiple field hospitals for the care of COVID-19 patients, which would effectively free up remaining tertiary, university, government and private hospitals to work at full capacity in the care of non COVID-19 patients.

## Provenance and peer review

Not commissioned, externally peer-reviewed.

## Funding

The authors have not declared any grant for this work from any funding authority.

## Disclosure

The authors have no financial ties or conflicts of interest to disclose.

## Registration of research studies

1Name of the registry: Ahmad Al Omari.

2Unique Identifying number or registration ID: 6382.

Hyperlink to your specific registration (must be publicly accessible and will be checked): https://www.researchregistry.com/browse-the-registry#home/

3.
